# Flora Medica: A Botanical Account of All the More Important Plants Used in Medicine, in Different Parts of the World

**Published:** 1839-04

**Authors:** 


Art. VII.—*Flora Medica: a Botanical Account of all the more important Plants used in Medicine, in different Parts of the World.* By John Lindley, ph.d. f.r.s., Professor of Botany in University College, London, &c.—*London*, 1838. 8vo. pp. 656.

In this work Dr. Lindley has undertaken to give a correct botanical description of the various species of plants which are used medicinally in different parts of the world. To this he has added the synonyms of other writers, and the vernacular names by which the plants are commonly known to the inhabitants of the countries where they grow.

The manner in which this task has been accomplished is such as we expected from a writer of such acknowledged botanical eminence as Dr. Lindley: the descriptions are clear; the language is in accordance with the existing state of the science; and the specific characters are given in considerable detail: all the parts of the plant, as stem, leaves, and flowers, being particularly described, so that mistakes cannot easily be made. But, while we thus approve of the execution of the work, we must regret with the author (as stated in his preface) that such a book has not appeared from the pen of a writer who is a medical man, and as well a pharmacologist as a botanist: for a more detailed account of the medical properties and active principles, with a more frequent mention of the diseases in which the different medicines have been advantageously given—in fact, the addition of a little materia medica to the botanical descriptions, would have rendered the work more valuable, particularly to one class of readers, for whose use it was chiefly designed, i. e. medical students. The medical properties of the plants are often very briefly alluded to, and in some instances entirely omitted: thus, when speaking of the *Papaver somniferum*, after detailing the specific characters, the author adds, "From the wounded half-ripe capsules flows a juice which concretes into opium, the well-known powerful narcotic drug." The uses of this important medicine are thus briefly alluded to, without a word being said of the chemical principles on which the narcotic properties depend. In a long article on the *Cinchona*, not a word is said of the properties of the bark: it is taken for granted that these are well known to all readers; but a few words might well have been added on the peculiar principles contained in each species or variety, as these are known to vary in the different kinds, adapting one more than another for administration in different forms of disease.

With these imperfections, however, the Flora Medica is a work of considerable value, and will be found particularly useful in clearing up the conflicting statements which have been made respecting the sources from which many medicinal plants, often of the commonest kind, are derived. By collecting a great mass of evidence, Dr. Lindley has either been enabled to arrive at a correct knowledge of the disputed species, or to show that the suppositions of various writers are erroneous; and that the source of the article is either unknown, or that the drug is procured indiscriminately from several different plants. For instance, the species of *Rheum* from which the real rhubarb of commerce is obtained is involved in much obscurity: it has been said by different authors to be the root of Rheum palmatum, R. undulatum, and R. emodi; and though, as Dr. Lindley says, "the opinion that Rheum palmatum is the source of the true officinal rhubarb continues to be generally entertained," yet it seems very probable "that the officinal drug obtained in the heart of Thibet, whither no botanist has ever penetrated, is the produce of some species still unknown."

In this, as in other doubtful cases, the author has given the botanical characters of all the known species of the plant. A very detailed account of the genus *Cinchona* is given, "to determine, if possible, the real origin of the barks known in trade and used in the shops:" but this seems scarcely possible; for the botanical distinctions between the species themselves are as much embroiled as the knowledge of whence the different varieties and finest qualities of bark are procured. Dr. Lindley describes twenty-four species, and states, as the result of his investigation, that the *pale barks* are chiefly obtained from Cinchona condaminea and C. micrantha; the *yellow bark* principally from C. lanceolata; and the *red bark* from a species not yet ascertained. The trees said to produce the pale and yellow barks in the London Pharmacopœia do not yield that which is employed in practice, and the bark procured from them is so inferior as to be valueless in pharmacy. The C. oblongifolia, to which the red bark is referred, on the authority of Mr. Lambert, yields a bark the nature of which is entirely unknown.

In the plan of this work, the genera are arranged according to their natural families, the sequence of the orders corresponding to that in the "natural system" of the author; but the work is so printed that the different orders may be separated, and rearranged at the pleasure of the professor. In conclusion, we must say that we have derived much pleasure and information from this volume; and we sincerely recommend it to those engaged in teaching botany and materia medica, to whom it will be highly useful as a book of reference. We fear that the generality of medical students have hardly time to make themselves acquainted with the botanical characters of all the medical plants; but those whose destiny it may be to practise their profession in foreign climates will find the Flora Medica a most useful companion.

